# Association between Light-Induced Dynamic Dilation of Retinal Vessels and Echocardiographic Parameters of the Left Ventricular Function in Hypertensive Patients

**DOI:** 10.3390/medicina56120704

**Published:** 2020-12-17

**Authors:** Małgorzata Peregud-Pogorzelska, Małgorzata Zielska, Miłosz Piotr Kawa, Katarzyna Babiak, Krzysztof Safranow, Bogusław Machaliński, Anna Machalińska

**Affiliations:** 1 Department of Cardiology, Pomeranian Medical University in Szczecin, 70-111 Szczecin, Poland; m1peregud@gmail.com (M.P.-P.); malzielska@gmail.com (M.Z.); 2 Department of General Pathology, Pomeranian Medical University in Szczecin, 70-111 Szczecin, Poland; milosz.kawa@pum.edu.pl (M.P.K.); machalin@pum.edu.pl (B.M.); 3 First Department of Ophthalmology, Pomeranian Medical University in Szczecin, 70-111 Szczecin, Poland; zpatog@pum.edu.pl; 4 Department of Biochemistry and Medical Chemistry, Pomeranian Medical University in Szczecin, 70-111 Szczecin, Poland; chrissaf@mp.pl

**Keywords:** hypertension, retinal microvascular imaging—RVA, dynamic vessel analysis—DVA, left ventricular systolic and diastolic dysfunction, echocardiography, endothelial dysfunction, microvascular disease, heart failure with preserved ejection fraction

## Abstract

*Background and Objectives*: The goal was to evaluate the association of dynamic retinal vessel analysis (DVA) with echocardiographic parameters assessing systolic and diastolic function of the left ventricle in hypertension (HT) patients with preserved left ventricle ejection fraction. *Materials and Methods*: This observational retrospective study recruited 36 patients with HT and 28 healthy controls. Retinal vessel diameter and reactions to flicker light were examined. Each patient was examined with echocardiography to assess left ventricular systolic and diastolic function. *Results*: Multivariate analysis revealed that hypertension was an independent factor associated with lower flicker-induced arterial vasodilatation (β = −0.31, *p* = 0.029). In the HT group, there was a significant positive association between left ventricular ejection fraction and flicker-induced arterial vasodilation (Rs = +0.31, *p* = 0.007). Additionally, end-diastolic left ventricular diameter negatively correlated with both arterial (Rs = −0.26, *p* = 0.02) and venous (Rs = −0.27, *p* = 0.02) flicker responses. Additionally, the echocardiographic characteristics of the left atrium (LA) remodeling in the course of HT, including the area of the LA and its antero-posterior dimension, were both negatively correlated with the arterial flicker response (Rs = −0.34, *p* = 0.003; Rs = −0.33, *p* = 0.004, respectively). From tissue Doppler parameters, the left ventricular filling index E/e’ negatively correlated with AVR (arteriovenous ratio) values (Rs = −0.36, *p* = 0.002). *Conclusions*: We revealed that systolic and diastolic function of the left ventricle in hypertensive patients is associated with retinal microvascular function.

## 1. Introduction

Endothelial dysfunction is one of the well-recognized key pathomechanisms responsible for hypertension (HT). Previous studies have demonstrated that cardiovascular disorders (CVDs) are linked with similar neurohumoral factors and microvascular endothelial dysfunction is important for CVD progression. The endothelial dysfunction in the coronary vessels is, to a large extent, related to the severity and prognosis of CVD [1].

The retinal microcirculation is an accessible vascular bed for the assessment of microvascular function [2,3]. Retinal vessel analysis (RVA) offers a unique possibility for the noninvasive examination of small vessels and is proposed to serve as a “window” for the investigation of endothelial function. Dynamic retinal vessel analysis (DVA) is a relatively new method that allows for the analysis of changes in the diameter of the retinal vessels in response to stimulation with flickering light via nitric oxide (NO) signaling and thus for the examination of endothelial dysfunction [4]. Recent studies indicated that NO is an important trigger of a cascade of different events that regulate vascular tone through the local release of several vasoactive agents that may initiate a signaling network resulting in retinal arterial vasodilation [5]. The reactivity of the retinal vessels under the influence of light flickering has already been compared previously with other dedicated methods to measure systemic endothelial function, such as flow-mediated dilatation (FMD) or pulse wave velocity (PWV) [6], however, these techniques are more demanding to perform. In this notion, the usage of continuous live image analysis allows for the observation of both retinal arteries and veins in their natural dynamic state. Previous studies on DVA have focused more on retinal vascular changes in patients with atherosclerotic disease focusing on the systemic nature of endothelial dysfunction [4]. However, only very few studies exist that investigated the association of DVA and cardiovascular disorders and cardiovascular risk factors in relation to endothelial dysfunction [6].

Endothelial dysfunction is an early pathophysiologic feature in the course of hypertension, and it is known that HT induces profound effects on both the structure and function of the microvasculature. Likewise, retinal microvessel changes, in particular generalized arterial narrowing and arteriolar-venous nicking, appeared not only to reflect current blood pressure levels but also blood pressure levels in the past [7,8] Moreover, reduced retinal hemodynamic response to flicker light has been reported in patients with systemic HT, reflecting long-term alterations of the retinal microvasculature [9]. Previously, we have also demonstrated a reduction in both retinal artery and vein responses to flicker light stimulation in patients with HT without heart problems compared with healthy controls [10]. Moreover, we revealed significant independent correlations between flicker-induced retinal microvasculature dilatation and several plasma biomarkers typical for endothelial dysfunction in the group of hypertensive patients [10].

Hypertension potentially leads to myocardial diastolic dysfunction and long-lasting atrial remodeling from chronic pressure overload. To date, there is very little knowledge about impaired endothelial vasodilatation of retinal vessels under clinical and subclinical CVD-related pathophysiological conditions, particularly the myocardial dysfunction in the course of HT-associated target organ damage. Therefore, the aim of this study was to analyze the relationship between the functional retinal microcirculatory response and selected echocardiographic parameters evaluating the systolic and diastolic left ventricle function in a group of hypertensive patients compared to healthy controls. In this study scenario, we examine whether defective diastolic function of the left ventricle is associated with HT-induced endothelial dysfunction in the vascular system.

## 2. Materials and Methods

### 2.1. Enrolment to the Study

The study group consisted of patients diagnosed with hypertension (36 patients, comprising 20 men and 16 women) treated at the Department of Cardiology, Pomeranian Medical University in Szczecin. The control group consisted of 28 age-matched apparently healthy volunteers (8 men, 20 women), who were referred to hospital in order to receive routine healthcare with no history of cardiovascular diseases, with normal blood pressure values during longitudinal measurements at home and correct blood pressure measurements at the hospital during the whole study.

Patients with coexisting ocular disease affecting the results of fundoscopy, i.e., glaucoma, advanced cataract, retinopathy, ocular inflammation and those treated with ocular surgery within 3 months prior to the study were excluded. We also excluded patients with systemic or chronic vascular diseases (rheumatological diseases, cancer, diabetes and history of stroke), Patients with valvar disease, previous myocardial infarction, or peripheral vascular disease were excluded from the study. Prior to the examination, informed written consent was obtained from all patients. The study protocol was approved by the Bioethics Committee of the Pomeranian Medical University in Szczecin and was compliant with the requirements of the Helsinki Declaration.

### 2.2. Examination of the Antropometric Parameters

All patients were assessed for body mass index (BMI) (according to the formula: body weight (kg)/body height (m)^2^) and waist-hip ratio (WHR) (waist circumference/hip circumference (cm)).

### 2.3. Examination of the Systemic Disease Status and Blood Pressure Measurement

Information on the disease, therapy, dyslipidemia and cigarette smoking (current and past) was obtained based on the patient’s interview, results of laboratory tests and analysis of medical records. Cigarette smoking was quantified in pack-years (calculated based on the mean number of cigarettes smoked per day declared by the patient and the number of smoking years). All patients were also asked about their level of physical activity. Each patient declared the total number of hours he or she spent on physical activity and its frequency during the week.

Before starting the blood pressure measurement, the patient rested for minimum 15 min. During the examination, blood pressure (BP) was measured by measuring blood pressure three times in five-minute intervals. The mean of three measurements was included in the study. From the obtained BP data, the systemic mean arterial pressure (MAP) was calculated as follows: MAP = diastolic BP + 1/3 (systolic BP−diastolic BP) mmHg.

### 2.4. Biochemical Parameter Examination

The concentration of creatinine in blood serum (glomerular filtration rate (GFR) was calculated based on the Cockcroft-Gault formula, with a normal level accepted as GFR > 60 mL/min) and fasting blood glucose (abnormal concentration above 100 mg/dL) were measured in each examined subject.

### 2.5. Ophthalmological Examination

Ophthalmological examination of both eyes, including the measurement of intraocular pressure (IOP) and fundoscopy, was done in each patient. All measurements of the retinal vessels were taken after full pupil dilation induced by the application of 1% tropicamide. Retinal vessels were evaluated in the next stage of the study. The mean value measured for both eyes was used for further analysis.

### 2.6. Static Retinal Vessel Analysis

**Figure 1:**
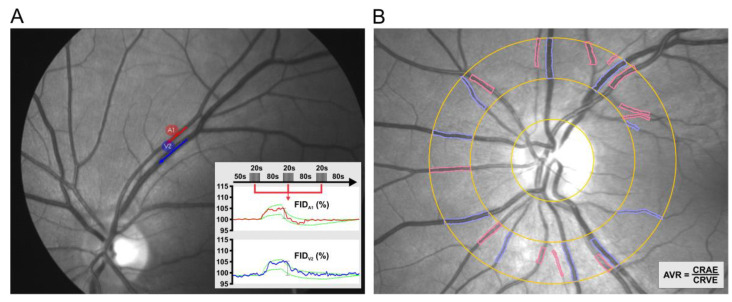
Representative outlook of the retinal vessel analysis (RVA) that contains two components. Dynamic retinal vessel analysis (DVA) (**A**), a dynamic measurement of flicker-induced dilatation (FID) of retinal arterioles (red) and venules (blue) using a standardized flicker protocol and Static vessel analysis (SVA) (**B**), a static measurement of arteriolar and venular diameters using a semi-automatic procedure. The arteriovenous ratio (AVR) is then calculated from the central retinal artery and vein equivalents measured independently (CRAE and CRVE, respectively).

Static vessel analysis (SVA) was done with a FF450 fundus camera (Zeiss AG, Jena, Germany). The obtained retinal images were analyzed using VISUALIS (version 2.61) and VesselMap (version 3.10) software (IMEDOS Systems, Ltd., Jena, Germany). Standard parameters included central retinal artery equivalent (CRAE), referring to the diameter of the central retinal artery, and central retinal vein equivalent (CRVE), referring to the diameter of the central retinal vein, as well as arteriovenous ratio (AVR), which is the quotient of CRAE/CRVE (Figure 1).

### 2.7. Dynamic Retinal Vessel Analysis

In the subsequent stage, a dynamic retinal vessel analysis (DVA) was performed as previously described in detail by Machalinska et al. [11]. The arterial and venous segments were selected based on the following criteria: no intersection or bifurcation in the measured segment, vascular curvature not exceeding 30°, distance from adjoining vessels equal to at least one vessel diameter and sufficient contrast with the surrounding fundus. The standard program for light stimulation (in DVA) consisted of three consecutive flicker cycles (flicker frequency 12.5 Hz with an 80 s break interval). The total test time was 350 s. The response was measured as the difference between the mean vessel diameter in the last 10 s of flickering stimulation and the mean vessel diameter for 30 s immediately before this stimulation, divided by the latter value. The results were expressed as the mean calculated for three flicker cycles (Figure 1). One artery and one vein were measured in each eye.

### 2.8. Echocardiography

Each patient had echocardiography performed by the same operator using a Vivid 7 Pro (GE Vingmed) apparatus connected to a 2.5–3.5 MHz phase probe. All measurements were averaged for 3 cardiac cycles. Left ventricular mass (LVM), left ventricular end-diastolic dimension (LVEDD), left ventricular end-systolic dimension (LVESD), posterior wall thickness (PWT), interventricular septal thickness (IVST) during diastole, the aortic bulb diameter and the size of the left atrium were measured in M-mode from the parasternal long-axis view according to the standards of the American Society of Echocardiography. Ejection fraction (EF) of the left ventricle was calculated based on left ventricular end-diastolic volume, and left ventricular end-systolic volume was measured from the apical two- and four-chamber view according to Simpson’s rule. LV mass as found on echocardiography was indexed (LVMI) using body surface area for each subject. Left atrial volume was calculated using the prolate ellipse method and indexed to body surface area (left atrial volume index, LAVI). The left ventricular diastolic function was evaluated using the mitral flow velocity. We measured the peak early-diastolic velocity (E), the peak end-diastolic velocity (A), the E/A ratio (early-diastolic to end-diastolic velocity), isovolumic relaxation time (IVRT), Tei index (myocardial performance index) and mitral early flow propagation velocity. We used tissue Doppler echocardiography (TDE) to identify the s’ wave corresponding with the systolic myocardial tissue velocity of the left ventricle (LV), the e’ wave corresponding with the diastolic myocardial tissue velocity during the rapid filling phase of LV, the a’ wave corresponding to the diastolic myocardial tissue velocity during atrial contraction and the e’/a’ ratio at two points (medial in the interventricular septum and lateral on the wall of the left ventricle). We also calculated the ratio of the mitral flow velocity to mitral annular velocity at the early diastolic phase (E/e’).

### 2.9. Statistical Analysis

Quantitative parameters measured in both eyes were averaged before further analysis. Since the distribution of the quantitative variables was, in most cases, significantly different from normal distribution, the nonparametric Mann-Whitney test was used to compare their values between groups and Spearman’s rank correlation coefficient was used to measure the strength of associations between them. Fisher’s exact test was used to compare qualitative variables between groups. Multivariate analysis of independent factors associated with retinal vessel parameters was performed with the general linear model (GLM). The statistical significance level was adopted at *p* < 0.05. The analysis was carried out separately for patients with and without diagnosed hypertension. The statistical power of the study was sufficient to detect with 80% probability associations between variables with an effect size corresponding to a correlation coefficient of ± 0.45 in the study group (36 patients) and ± 0.50 in the control group (28 subjects).

## 3. Results

The clinical characteristics of the patients are presented in Table 1. The study and control groups were matched for age and all participants completed the study. There were no significant differences between patients and controls for the level of physical activity, mean arterial blood pressure or history of cigarette smoking. Dyslipidemia and current smoking status were more common in hypertensive subjects. Values of body mass index (BMI) and waist-hip ratio (WHR) were significantly higher in the hypertensive group compared to those in controls. There were no significant differences in the level of creatinine and glomerular filtration rate (GFR) between groups, whereas the fasting glucose level was significantly higher in hypertensive patients (none of the patients or controls were previously diagnosed with diabetes).

**Figure 2:**
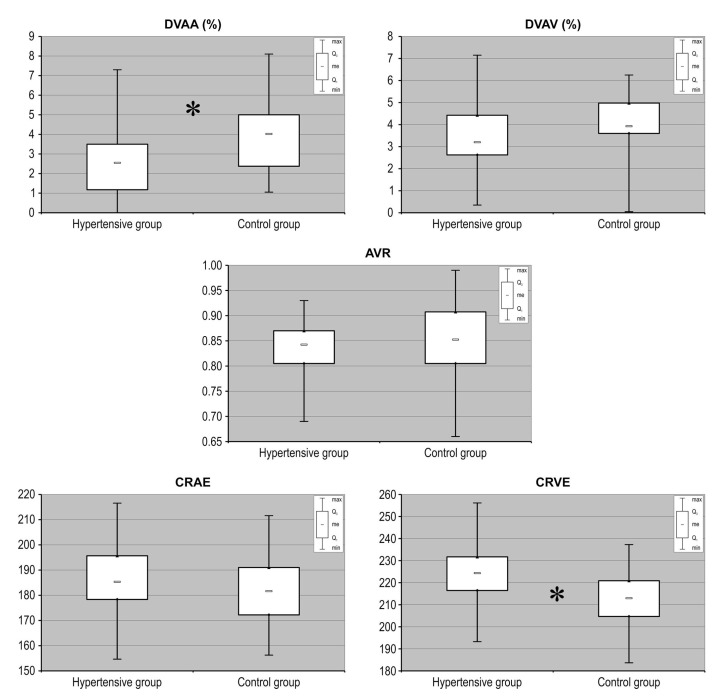
Measurement of selected retinal vessel parameters (AVR, CRAE, CRVE, DVAA, DVAV) in patients with hypertension recruited to the study and compared with matched control subjects without hypertension. AVR: arterio-to-venous ratio); CRAE: central retinal artery equivalent; CRVE: central retinal vein equivalent; DVAA: dynamic vessel analysis of arteries; DVAV: dynamic vessel analysis of veins. Data are shown as median, quartiles, interquartile range, minimum and maximum. * *p* < 0.01.

Table 2 provides the values of the retinal vessel parameters obtained for both investigated groups. There were no participants with missing data for each variable of interest. We observed that the arterial response to flicker stimulation was lower in hypertensive patients compared with that in the controls (*p* = 0.005). Multivariate analysis of patients and controls adjusted for age, sex, LVMI, BMI and dyslipidemia revealed that hypertension was an independent factor associated with lower flicker-induced arterial vasodilatation (β = −0.31, *p* = 0.029), while no significant association was found for any of the covariates. No significant differences in venous response to flicker stimulation were identified between groups (*p* = 0.086). Similarly, the arterial-to-venous ratio (AVR) obtained by static vessel analysis did not differ between analyzed groups of eyes. The evaluated retinal vessel responses in hypertensive patients compared to healthy subjects are presented in Table 2. In addition, after calculation of the central retinal artery (CRAE) and vein (CRVE) equivalents, we observed that the average static width of retinal venules is significantly greater in the group with HT compared to their matched normotensive controls (Figure 2).

Table 3 shows the echocardiographic characteristics obtained in hypertensive patients and controls. There were no participants with missing data for each variable of interest. In patients with hypertension, echocardiography analysis showed a significant enlargement of the left atrium in its basal antero-posterior dimension (*p* = 0.039), area (*p* = 0.014) and volume (*p* = 0.031) compared to those in controls (Table 3).

Subsequent correlation analysis of the echocardiographic parameters with retinal vessel characteristics showed positive association between left ventricular ejection fraction values and flicker-induced arterial vasodilatation (Rs = +0.31, *p* = 0.007 for hypertensive patients and Rs = +0.35, *p* = 0.009 for controls). In hypertensive patients, end-diastolic left ventricular diameter (LVEDD) negatively correlated with both the arterial (Rs = −0.26, *p* = 0.02) and venous (Rs = −0.27, *p* = 0.02) responses to stimulation with flicker light. No such correlation was found in the control group. Isovolumic relaxation time was negatively correlated with flicker-induced venous vasodilatation (Rs = −0.32, *p* = 0.005) in patients with arterial hypertension, and there was no such correlation in the control group (Rs = +0.01, *p* = 0.92).

Next, we analyzed the echocardiographic markers of the left atrium remodeling in the course of HT, such as its size and geometry, I In hypertensive patients, the area of the left atrium (LA) negatively correlated with the flicker responses of the retinal arteries (Rs = −0.34, *p* = 0.003) and retinal veins (Rs = −0.25, *p* = 0.03). In the control group, the opposite correlation was observed (Rs = +0.34, *p* = 0.009 for the retinal arteries and Rs = −0.007, *p* = 0.95 for retinal veins). Likewise, the arterial response to stimulation with flicker light negatively correlated with the antero-posterior LA dimension (Rs = −0.33, *p* = 0.004) in patients with arterial hypertension. Interestingly, in the control group, the opposite correlation was observed (Rs = +0.34, *p* = 0.01). The volume of the left atrium (indexed to the body surface area) in the group with arterial hypertension was negatively correlated with the venous response to flicker stimulation (Rs = −0.23, *p* = 0.04), and no correlation was found in the control group.

The parameter of tissue Doppler imaging, such as the E/e’ ratio, significantly negatively correlated with AVR values (Rs = −0.36, *p* = 0.002) in the hypertensive group, and no correlation was observed in the control group.

Remarkably, there was no significant difference between DVA in patients taking drugs that block the renin-angiotensin-aldosterone system and those who did not take these drugs.

To more accurately characterize the effect of the selected cardiac and body parameters and blood pressure on DVA results, we evaluated the association between DVA and cardiac parameters and body morphometric characteristics, such as BMI and WHR, as well as systolic and diastolic and mean blood pressure. We aimed to investigate whether general body parameters in hypertensive patients are linked with retinal vascular characteristics. The results of such detailed analysis are displayed in Table 4.

## 4. Discussion

It was found that the prevalence of HT is increasing globally and that, currently, it is estimated that more than 25% of the worldwide population is hypertensive [12]. HT contributes to damage to several organs, resulting in kidney failure, left ventricular hypertrophy and subsequent dysfunction and large vessel wall remodeling, producing local distension or aneurysms [13,14,15]. HT is also known to have profound effects on both the function and structure of the microvessels [16,17]. Inflammation, oxidative stress and endothelial dysfunction have been hypothesized to be key mechanisms that provide the background for the development of HT and its target organ complications [18,19]. The decreased bioavailability of nitric oxide (NO), as well as increased proinflammatory and oxidative stress-related molecules, play a crucial role in contributing to the decrease in endothelial vasodilative properties [20,21]. NO produced by the endothelium induces vasodilation but also has an anti-atherosclerotic effect by inhibiting the interaction between platelets, leukocytes and the vessel wall components and inhibiting the proliferation of smooth muscle cells.

Endothelial dysfunction plays a role in the pathogenesis of cardiovascular disease and retinal vasculature may provide a non-invasive approach to examine the microvascular endothelial function.

Hypertension can lead to myocardial diastolic dysfunction [22,23]. Left ventricular diastolic function is determined by many echocardiographic parameters [22]. The most important parameters include: indexed volume of the left atrium, left ventricular mass index and Doppler and tissue Doppler parameters.

The echocardiography analysis of hearts in our HT patients showed an enlargement of the left atrium compared to controls, and this may indicate the presence of left ventricle diastolic dysfunction in hypertensive patients. In echocardiography, the left atrial volume serves as a morphophysiologic expression of left ventricular diastolic dysfunction, and LA enlargement is associated with adverse cardiovascular outcomes [24]. Atrial enlargement is often diagnosed in hypertensive patients [25] and has been suggested as a marker of the severity and duration of diastolic dysfunction [26]. Interestingly, the area of the left atrium and the anteroposterior dimension of the left atrium negatively correlated in HT patients from our study with retinal dynamic arterial reactivity, and the correlation between retinal vascular reactivity and left ventricular mass was also negative, which may indicate reduced reactivity of the retinal vessels in people with an enlarged mass of the left ventricle and size of the left atrium. Impaired arterial and venous retinal response to flicker light stimulation negatively correlated with end-diastolic left ventricular diameter, isovolumic relaxation time, area and antero-posterior dimension of the left atrium, all of them being indirect markers of myocardial diastolic dysfunction in the left ventricle. Finally, the ratio of E/e’ also negatively correlated with AVR values, also indicating the influence on the structural changes in the vascular system of the retina, including longitudinal arterial narrowing and venular widening. This result may also indicate that the presence of left ventricle diastolic dysfunction in hypertensive patients is accompanied by structural adaptation in retinal arteries and veins, expressed by low AVR values. Our present study also revealed the correlation between left ventricular ejection fraction and flicker-induced arterial vasodilatation measured with a dynamic retinal vessel analyzer (DVA)-based approach in hypertensive patients and healthy subjects. This may indicate that the increase in left ventricular ejection fraction is accompanied by a better dynamic functionality in distant vascular beds, including retinal arterioles. Altogether, all the obtained results from this study strongly indicate the impaired reactivity of retinal vessels in hypertensive subjects with compromised left ventricular diastolic function. On the other hand, the left ventricular mass (indexed to body surface area), which is a well-documented risk factor for cardiovascular diseases, including primary and secondary HT [27], was considerably greater in the hypertensive group in our study. Furthermore, in the hypertensive group, the correlation between the reactivity of the retinal vessels and the indexed mass of the left ventricle was negative, which may indicate a decreased vascular reactivity in patients with a greater mass of the left ventricle. Likewise, LV mass was previously correlated with structural changes in the cardiovascular system under physiological conditions in sport athletes [28] and pathological conditions including end-stage renal disease [27].

According to the guidelines of the American and European Societies of Echocardiography, the diastolic function is evaluated among other things based on the spectrum of mitral valve flow (MVF) and tissue Doppler parameters [29]. The term diastolic function encompasses active myocardial relaxation, which significantly influences early ventricular filling, as well as passive end-diastolic ventricular stiffness, which impacts late ventricular filling. Different echocardiographic parameters assess different aspects of diastolic left ventricle function, its consequences and determinants [29]. Since there is no single parameter indicated that would allow a simple assessment of the myocardial diastolic function, several echocardiographic measurements should be taken and conclusions drawn based on them by an experienced cardiologist. Although echocardiography is immensely valuable in the noninvasive evaluation of human myocardial diastolic function, this technique is a time-consuming medical exam, which is highly subjective and depends on the skills of the person who is performing this examination. The main limitation of echocardiography, however, is that the recording of reliable echocardiography images can be technically difficult if a skilled echocardiographer is not available at the time of the diagnostic examination of the patient with potential left ventricle insufficiency, especially at an emergency ward in a small local or district hospital.

Some conditions predispose patients to myocardial diastolic dysfunction, including systemic hypertension, diabetes mellitus and myocardial ischemia. Endothelial dysfunction has been found to be related to impaired left ventricular diastolic reserve and subsequent diminished exercise capacity [30] Vascular dysfunction of the endothelium is also nowadays an early sign of pro-inflammatory atherosclerosis that can be detected before coronary heart disease or other vascular complications occur in the body. Principally, endothelial dysfunction can be involved in the pathophysiology of cardiac abnormalities, as in the coronary vasculature it can lead to microvascular coronary dysfunction. Finally, endothelial dysfunction has been demonstrated to be a hallmark of all stages of cardiovascular disease, from the prevention of atherosclerosis to treating different cardiac pathologies [31,32]. Lately, only one study has demonstrated the correlation between DVA and diastolic function indexes in patients with heart failure due to coronary artery disease [33] but not in patients with diastolic dysfunction in the course of isolated hypertension. As one of the causes of cardiovascular diseases, including diastolic dysfunction, is endothelial dysfunction [34], therefore, there is much interest in new, automatic or semiautomatic and less skill-dependent techniques to assess endothelial impairment in screened patients. The dynamic analysis of retinal micro-vessels may provide details on vascular function and appears to be a direct noninvasive method to study the endothelial status in retinal vessels in the course of CVD. Moreover, DVA can be performed by a trained nurse or a person with middle-level medical education, such as an electroradiology technician.

In the past, several authors revealed the potential of DVA examination to specify the subjects with metabolic abnormalities, such as hypercholesterolemia [35] or hyperglycemia as well as patients with diabetes [36] and heart failure [6] by measuring the microvascular endothelial dysfunction in their retinas. In particular, the changes in the reactivity of retinal arterioles to flicker light appeared to be predictive of the presence of several cardiac abnormalities, including coronary artery disease and heart failure [33]. Importantly, it was proposed that DVA may be treated as “the window to the heart”, discriminating patients with some abnormal cardiac functions from healthy subjects. In this notion, our study demonstrated, for the first time to our knowledge, that a potential relationship exists between the diastolic left ventricular echocardiographic parameters and dynamic retinal vessel functionality in hypertensive patients.

The retina has been used as a surrogate to study general vascular changes. There is a growing interest in the identification of retinal microvascular changes as a safe, easily accessible, low cost and time-efficient approach to improve our understanding of the vascular complications associated with HT. There is evidence that microcirculation is coupled with macrocirculation not only in terms of structural but also functional parameters. For example, it was found that increased aortic stiffness occurring in hypertension was associated with microvascular remodeling. Central and brachial systolic, diastolic and mean BPs were significantly correlated with lumen diameter and the outer diameter of retinal arterioles [37] In addition, in a subgroup with cardiac dysfunction, the central and brachial pulse pressures were positively associated with retinal wall thickness, wall cross sectional area and wall to lumen ratio. Furthermore, retinal microvasculature was found to be globally affected in severe diabetic retinopathy. It was found that in eyes with poorer visual acuity in the course of diabetic retinopathy, there was lower CRAE and dimensions of arterioles and venules [38] This indicates that a reduction in DVAA may result from a decreased retinal capillary blood flow due to alterations in vascular structure in the course of chronic vascular-related complications. The patient’s old age, female sex, obesity, arterial hypertension and left ventricular hypertrophy predispose them to the development of myocardial diastolic dysfunction [23]. In our analysis, we took into account the above factors. In particular, hypercholesterolemia is associated with endothelial dysfunction, which is potentially reversible with lipid-lowering therapy. Moreover, systematic studies on flicker-induced retinal vasodilatation in hypercholesterolemia previously showed a substantial retinal microvascular dysfunction in such a group of patients [35].

We acknowledge that this study has several limitations, including the fact that the studied population was relatively small. Thus, this study should be treated as a small-population pilot study. Furthermore, this study included only Caucasian subjects and therefore our conclusions may not be generalizable to people of other races.

Altogether, DVA might represent an adjunct and clinically relevant microvascular alternative outcome measure that can be used in a clinical approach for HT diagnosis and therapy. The guidelines of the European Society of Cardiology recommend examination of the fundus in people with hypertension of the second and third degree. However, due to the frequency of HT in general society, ophthalmologic examination should be considered in all hypertensive patients [22]. Standard fundus examination then could be extended to DVA analysis. DVA is not proposed as a substitute for echocardiography, but it could be a more direct and simple test for the detection of endothelial dysfunction, initiating further cardiovascular testing when indicated.

## 5. Conclusions

To our knowledge, there have been no previously published reports on associations between echocardiographically measured parameters of the diastolic cardiac dysfunction and retinal vasodilatory response as a marker of microvascular endothelial dysfunction in hypertensive patients. On the basis of the obtained results, we propose that the DVA method might be useful for the detection of the early phases of myocardial diastolic dysfunction as a potential alternative microvascular outcome measure in patients with diagnosed HT undergoing longitudinal medical observation for this chronic disease. However, further studies in this area are necessary.

## Figures and Tables

**Table 1:** Clinical characteristics of the study groups (abbreviations explained in the text).

Parameter	Hypertensive Patients (*n* = 36)	Control Subjects (*n* = 28)	*p* Value
Sex (men/women)	20/16	8/20	0.043
Age (years) (mean ± SD)	57.14 ± 9.00	53.63 ± 6.47	0.10
BMI (kg/m^2^) (mean ± SD)	29.43 ± 4.69	26.07 ± 4.15	0.005
WHR (ratio) (mean ± SD)	0.95 ± 0.10	0.84 ± 0.08	0.00002
Systolic blood pressure (mmHg) (mean ± SD)	124.86 ± 13.90	125.42 ± 14.93	0.866
Diastolic blood pressure (mmHg) (mean ± SD)	83.55 ± 8.67	84 ± 9.25	0.887
Number of patients taking RAASinhibitors (*n*) (%)	25 (69.44)	-	
Number of patients taking a beta blocker (*n*) (%)	19 (52.77)	-	
Number of patients taking diuretics (*n*) (%)	2 (5.55)	-	
Number of patients taking calcium channel blockers (*n*) (%)	2 (5.55)	-	
MAP (mmHg) (mean ± SD)	97.50 ± 9.55	97.93 ± 10.16	0.877
Currently smoking (*n*) (%)	13 (36.11)	3 (10.71)	0.023
Smoked in the past (*n*) (%)	13 (36.11)	3 (10.71)	0.130
Cigarette smoking (pack-years) (mean ± SD)	12.76 ± 13.31	10.21 ±19.36	0.142
Duration of hypertension (years) (mean ± SD)	8.03 ± 8.72	-	
Physical activity (hours/week) (mean ± SD)	2.91 ± 2.97	1.85 ± 2.66	0.183
Dyslipidemia (*n*) (%)	14 (38.88)	2 (7.14)	0.004
**Biochemical parameters of blood**			
Fasting glucose (mg/dl) (mean ± SD)	101.45 ± 10.30	93.56 ± 13.44	0.031
Creatinine (mg/dl) (mean ± SD)	0.81 ± 0.15	0.80 ± 0.14	0.599
GFR (ml/min/1.73^2^) (mean ± SD)	95.32 ± 11.94	88.93 ± 13.14	0.056

*n*: number of subjects; SD: standard deviation; BMI: body mass index; WHR: waist-hip ratio; RAAS (renin-angiotensin-aldosterone system); MAP: mean arterial pressure; GFR: glomerular filtration rate.

**Table 2:** Parameters of retinal vessels measured in the study groups.

Parameters of Retinal Vessels	Hypertensive Patients (*n* = 36)	Control Subjects (*n* = 28)	*p* Value
Arterial-to-venous ratio (AVR)	0.83 ± 0.05	0.85 ± 0.07	0.260
Central retinal artery equivalent (CRAE)	185.9 ± 12.8	187.9 ± 12.5	0.272
Central retinal vein equivalent (CRVE)	224.2 ± 13.9	213.5 ± 12.9	0.004
Dynamic vessel analysis of arteries (DVAA) (%) (mean ± SD)	2.51 ± 1.63	3.86 ± 1.82	0.005
Dynamic vessel analysis of veins (DVAV) (%) (mean ± SD)	3.52 ± 1.61	3.97 ± 1.39	0.086

**Table 3:** Echocardiographic characteristics of the study groups.

Echocardiographic Parameters	Hypertensive Patients (*n* = 36)	Control Subjects (*n* = 28)	*p* Value
Antero-posterior left atrium dimension (from the parasternal long-axis view in M-mode) (cm) (mean ± SD)	4.03 ± 0.52	3.75 ± 0.62	0.039
Left atrium area (cm^2^) (mean ± SD)	20.55 ± 4.06	18.12 ± 3.85	0.014
Left atrium end-diastolic volume (ml) (mean ± SD)	58.17 ± 16.37	50.57 ± 15.20	0.031
Left atrium maximum volume index (ml/m^2^) (mean ± SD)	29.24 ± 8.09	27.79 ± 15.20	0.315
Left ventricle ejection fraction (%) (mean ± SD)	59.30 ± 7.08	62.14 ± 4.17	0.112
Heart rate (bmp) (mean ± SD)	73.36 ± 11.16	74.92 ± 9.94	0.009
LV mass (g) (mean ± SD)	229.91 ± 73.77	194.83 ± 57.09	0.07
LV mass index (g/m^2^) (mean ± SD)	114.59 ± 33.06	105.51 ± 26.76	0.29
E/A ratio (mean ± SD)	1.09 ± 0.38	1.16 ± 0.39	0.480
E/e’ (mean ± SD)	9.66 ± 2.21	8.97 ± 2.46	0.158
e’ med (m/s) (mean ± SD)	0.07 ± 0.02	0.07 ± 0.01	0.930
e’ lat (m/s) (mean ± SD)	0.07 ± 0.02	0.08 ± 0.03	0.866
End-diastolic left ventricular internal diameter (cm) (mean ± SD)	4.76 ± 0.51	4.71 ± 0.56	0.919
Isovolumic relaxation time (IVRT) (ms) (mean ± SD)	101.27 ± 11.83	97.82 ± 11.07	0.302

*n*: number of subjects; LV: left ventricle; E/A: ratio of the early (E) to late (A) ventricular filling velocities; E/e’ ratio: mitral flow E wave velocity to mean e’ wave velocity at mitral annulus ratio; e’med.: e’ wave velocity on septal mitral annulus tissue Doppler; e’ lat: e’ wave velocity on lateral mitral annulus tissue Doppler; SD: standard deviation.

**Table 4:** Spearman’s correlation coefficients for retinal vessel analysis and cardiac and body parameters or blood pressure data in hypertensive patients in comparison with the control group.

	BMI	WHR	SBP	DBP	MAP
Hypertensive group					
AVR	−0.048	−0.194	−0.025	0.081	0.014
CRAE	0.058	−0.182	−0.027	−0.070	−0.083
CRVE	0.086	0.028	0.092	−0.118	−0.034
DVAA	* **−0.242** *	−0.007	0.043	* **0.256** *	0.148
DVAV	−0.008	−0.173	0.215	0.222	* **0.264** *
Antero-posterior left atrium dimension (cm)	* **0.507** *	* **0.498** *	0.144	0.160	0.205
Left atrium area (cm^2^)	* **0.356** *	0.129	−0.060	−0.184	−0.160
Left atrium end-diastolic volume (mL)	0.171	0.243	−0.176	−0.091	−0.109
Left atrium maximum volume index (mL/m^2^)	−0.080	0.012	−0.150	−0.174	−0.167
Left ventricle ejection fraction (%)	−0.170	* **−0.321** *	0.227	* **0.337** *	0.261
LV posterior wall thickness (cm)	*0.343*	*0.401*	0.137	0.089	0.161
Left interventricular dimension (cm)	0.252	* **0.419** *	0.171	0.287	0.289
Left ventricle dimension (cm)	0.256	0.306	−0.009	−0.039	−0.037
Left ventricle mass (g)	*0.449*	*0.508*	0.071	0.135	0.156
Left ventricle mass index (g/m^2^)	0.265	* **0.352** *	0.078	0.085	0.117
**Control group**					
AVR	−0.219	−0.250	*−0.531*	*−0.666*	*−0.656*
CRAE	−0.021	−0.210	*−0.503*	*−0.601*	*−0.618*
CRVE	0.220	0.022	−0.008	0.080	0.015
DVAA	0.047	0.123	0.083	0.280	0.196
DVAV	0.060	0.089	−0.194	−0.136	−0.181
Antero-posterior left atrium dimension (cm)	0.346	0.318	0.311	0.024	0.150
Left atrium area (cm^2^)	0.325	0.195	0.157	0.256	0.222
Left atrium end-diastolic volume (mL)	0.214	0.197	0.119	0.148	0.162
Left atrium maximum volume index (mL/m^2^)	−0.152	−0.150	−0.085	−0.075	−0.080
Left ventricle ejection fraction (%)	0.149	−0.164	−0.097	0.091	0.008
LV posterior wall thickness (cm)	*0.399*	*0.515*	*0.719*	0.314	*0.496*
Left intraventricular dimension (cm)	0.253	0.282	0.319	0.212	0.303
Left ventricle dimension (cm)	*0.457*	*0.534*	*0.495*	0.309	*0.419*
Left ventricle mass (g)	*0.447*	*0.456*	*0.686*	*0.403*	*0.582*
Left ventricle mass index (g/m^2^)	0.151	0.231	*0.607*	0.243	*0.455*

Statistically significant results (*p* < 0.05) are shown in red italics. The bold font corresponds to unique correlations, which were statistically significant only in patients with hypertension, not controls. BMI: body mass index; WHR: waist-hip ratio; SBP: systolic blood pressure; DBP: diastolic blood pressure; MAP: mean arterial pressure.
